# A Treasured Remembrance and Great Opportunity

**Published:** 1919-11-01

**Authors:** 


### The Hospital

The Workers' Newspaper of Administrative Medicine and Institutional Life, Administration, National Insurance and Health.

No. 1743, Vol. LXVII. SATURDAY, NOVEMBER 1, 1919. PRICE TWOPENCE

### A TREASURED REMEMBRANCE AND GREAT OPPORTUNITY.

In another column we have called attention to the services on All Saints' Day which are being held, not only in hospitals, but in places of worship almost everywhere. These services, it has been intimated, will be attended by large numbers of members of the medical profession and nursing sisters of all grades. These have, most of them, seen the horrors perpetrated in the course of the worst war of the world, and to them all are attached remembrances of the happenings in the recent past in connection with the supreme sacrifice made by some of the best men and women of our race in the hour of danger, when the very existence of the nation hung in the balance, and the outlook for the future was awe-inspiring and terrible. Should it not be possible for the Committees of Management to join with their staffs in rendering homage to the brave dead, and perpetuating their memory by gifts from the living to an amount adequate to meet the pressing and urgent claims for funds under which so many of our voluntary hospitals may labour at the present time? If these memories can be brought to unite all who share them in the cause of bringing succour to the sick in the Homeland who need such services, then it may be possible, and should, in fact, result, that no hospital of importance, metropolitan or provincial, large or small, should enter upon the New Year with the slightest cause for anxiety as to its future maintenance and the extension of its efficiency to every corner of the field which each may cultivate.

It would indeed be a novel and soul-inspiring decision for the managers of the hospitals everywhere to identify themselves with this splendid form of united action in memory of our nearest and dearest whom we have lost in the war. Everywhere there is the desire for commemoration and the celebration of the victory vouchsafed to the British nation and Empire, and of their deliverance from the awful dangers with which both were threatened in the recent past. No form of commemoration could surely be more popular than that here suggested, and none more likely to yield adequate fruits in personal service and material means, whereby the future financial outlook and extended efficiency of every establishment charged with the duty of succour for the sick should be strengthened, and all anxiety for the future in regard to these matters effectually removed. A long pull, a strong pull, and a pull altogether, will bring to the hospitals' treasuries ample funds at this time before the close of the year, providing those responsible for the business of our hospitals will awaken in time, enter heart and soul into the propaganda here suggested, and never rest until the results prove adequate to the needs of each institution wise enough to yoke the outpouring of gratitude and thanks by the people of all classes throughout the British Islands, in connection with the memorial services to those who have found immortality, on behalf of the living whose hearts must overflow with gratitude for God's great mercy extended to them as a people, a Nation and Empire in the year of Victory. The whole people are anxious, hopeful that some opportunity may be given to all classes to unite in some great effort which will mark the reality of their feelings in these great affairs, and seal in full measure the expression of their earnest desire to provide everywhere, adequately and fully, for the future care and succour of all their neighbours who in these anxious days are less fortunate in their surroundings than themselves.

As a final word may we say in all earnestness that we regret that the utterance attributed to Lord Knutsford, in which he ill repays the splendid liberality which has followed the issue by himself of many appeals for the voluntary hospitals, should be clouded by his loss of faith, as expressed in the declaration printed in the *Saturday Review* of

October 25, to the effect that "the necessity will come, if it has not come already," to put the voluntary hospitals on the rates? We know, and can produce figures to demonstrate, that the utterance is a feeble and ill-founded one, opposed to the will and wishes of the British people, though in a sense it might become as dangerous to the welfare and needs of the suffering amongst us as it is unwarranted by the financial position of to-day of the whole of the voluntary hospitals in these islands. The greatest danger, and in fact in our judgment the only danger, to the future financial prosperity of our great voluntary hospitals, the pride of the whole world, is feeble faith amongst the managers of these institutions, which may cause a large number of them to invite to the withholding of further voluntary gifts by an utterance like that ascribed to Lord Knutsford, which we hope, on consideration, he will take an early opportunity to explain away and withdraw.

